# Supplementary material for: Time to initiate trophic feeding and predictors among preterm neonates admitted at General Hospitals in Tigray, 2025
Source: PLoS One. 2026 Mar 23;21(3):e0335385. doi: 10.1371/journal.pone.0335385 (PMC13008079; doi:10.1371/journal.pone.0335385)
Supplement: Attachment — Submitted filename: Responce letter.docx [file pone.0335385.s002.docx]

Name: Teklebrhan Welderufael Kidane
 Address: Aksum
 Email Address:welderufaelteklebrhan@gmail.com
Phone Number: +251925065464
Date: October 25, 2025
***Dear: Erika Joy Florentino Macalalad***

**Subject: Response to Manuscript ID**: PONE-D-25-54861

I hope this message finds you well. I am writing in response to the comments and suggestions provided by the reviewers regarding my manuscript titled “Time to initiate trophic feeding and predictors among preterm neonates admitted at General Hospitals in Tigray, 2025.” I appreciate the opportunity to address their feedback and make improvements to my work.

**Summary of Revisions:**

**Editor Comment 1:** There are copying extracts from previous publications, especially outside the method section, word for word is unacceptable and revise the manuscript to rephrase the duplicated text and site all your sources.

**Response:**

I appreciate the reviewer’s feedback regarding the use of previously published materials. I have carefully reviewed the manuscript and made the following adjustments to address this concern:

I have rephrased all sections identified as containing verbatim extracts from prior publications, ensuring that all content is original and appropriately cited.

I have provided additional context and discussion to enhance the uniqueness of the content and to better integrate the relevant literature into the manuscript.

**2. Editor Comment 2**

Before we can proceed we noticed that you have provided two dates of recruitment .December 20,2024 to February 30,2025 and August 1,2024 to May 30,2025.please clarify and ensure that in the method section the day, month and year of the start and end of the recruitment for this study.

**Response:**

Thank you for your attention to detail regarding the recruitment dates. I appreciate the opportunity to clarify this matter.

The date range for the recruitment of data collectors was indeed from **December 20, 2024, to February 30, 2025**. The other date range, **August 1, 2024, to May 30, 2025**, pertains to the period from title selection to the completion of graduation, which is separate from the recruitment phase and I removed it from the manuscript.

I ensured that the methods section of the manuscript is revised to clearly reflect these distinctions, specifying the recruitment dates for data collectors accurately

**Editor Comment 3:**

Provide additional details regarding participant consent. In the ethics statement in the methods and outline submission information please ensure that you have specified.

**1.** Whether the consent was informed?

**2.**what type you obtained (written or verbal, and if verbal how was documented and witnessed ).if your study include minors ,state whether you obtained consent from the parents or guardians .if the need for consent was waived by the ethics committee ,please include this information.

**Response:**
Thank you for your valuable feedback regarding participant consent. I appreciate the opportunity to clarify this important aspect of our study.

**1. Informed Consent:** Yes, all participants provided informed consent prior to their involvement in the study.

**2. Type of Consent:** I obtained written consent from the parents or guardians of the preterm neonates involved in the study. The written consent forms were securely documented and stored and we maintained a record of this process.

In summary, since my study includes preterm neonates (considered minors), consent was obtained from their parents or guardians, ensuring they were fully informed about the study and its implications.

**Conclusion:**

I believe that the revisions made have significantly improved the quality of the manuscript. I appreciate the valuable feedback and hope that the changes meet the expectations of the reviewers and the editorial board.

Thank you for your consideration. I look forward to your response.

Sincerely,

Teklebrhan Welderufael Kidane
Aksum University
MSc, in pediatrics and child health
