# Supplementary material for: Time to initiate trophic feeding and predictors among preterm neonates admitted at General Hospitals in Tigray, 2025
Source: PLoS One. 2026 Mar 23;21(3):e0335385. doi: 10.1371/journal.pone.0335385 (PMC13008079; doi:10.1371/journal.pone.0335385)
Supplement: Attachment — Submitted filename: Comments to authors.docx [file pone.0335385.s004.docx]

**Manuscript title:** "Trophic Feeding Initiation and its Predictors in Preterm Neonates Admitted to Neonatal Intensive Care Units

- Preterm enteral nutrition feeding is an important topic with significant variability despite multiple recommendations. I applaud the authors’ efforts to analyze trophic feeding initiation.

**Abstract:**

- Introduction: in the abstract should be a short convictive statement showing the identified gap.
- Conclusion: which factors are risk and which are preventive should be concluded. Make the conclusion sound with specific recommendations.
- AIM and primary outcomes not defined
- Ethics: is written informed consent waved or is it signed? it should clearly be stated. If signed who signed the consent?

**Introduction:**

- The introduction appears vague and extensive. It should focus on what is known of the problem and what gaps this study looks to evaluate and solve.
- Line 62-67-paraphrase the idea
- Please describe, in a succinct way, the presence of feeding protocols at the hospitals
- Please summarize line 120-123 including reference citations
- The manuscript from line 129 -134 is not convective to conduct this study. Please revise it. What gaps were identified and what was your study’s main aim?
- What other studies found regarding your topic and what is left should be explained.
- Generally, introduction section should focus more on reasoning’s why providers do not start trophic feeds early despite the evidence available. Also, state how this manuscript will contribute to the evidence available regarding this topic.

**Method:**

- Please define pre-diagnosed Stage II or III NEC, Stage III asphyxia and why is this an exclusion criterion
- The Methods and materials section does not provide adequate information to replicate the study.
- Simple definitions are not included, like: how are TF provided?
- Line 227: Survival time is a confusing name for the time to start TF
- Line 234-246 the idea on this text should be clear and summarized: and also please specify/clarify the purpose of the questionnaire; to whom it was administered? This goes accordingly with my comment regarding lack of clarity about the execution of the study.
- Please omit line 274-278

**Result:**

Line 295-310 un-necessary redundant information since the information is provided with Table 1

Line 350-351 “Kaplan-Meier curves constructed to compare the overall survival patterns over time ‘’for’’ between different groups”. Please omit ‘’for’’

**Discussion:**

The discussion is focused on comparing the findings with those noticed in other studies. However, are the findings aligned with international literature?

Overall the discussion section needs major revision; does not clearly synthesize your findings and the implication of these findings.

The limitation section should be paraphrased

Regarding language: there are English grammatical and syntaxis errors in the whole document. Please ensure that, before resubmission, a person proficient in written English edits the manuscript.
