# Supplementary material for: Time to initiate trophic feeding and predictors among preterm neonates admitted at General Hospitals in Tigray, 2025
Source: PLoS One. 2026 Mar 23;21(3):e0335385. doi: 10.1371/journal.pone.0335385 (PMC13008079; doi:10.1371/journal.pone.0335385)
Supplement: Attachment — Submitted filename: Response to Reviewers.docx [file pone.0335385.s005.docx]

Response to Reviewers

: PONE-D-25-54861

Title: “Time to initiate trophic feeding and predictors among preterm neonates admitted at General Hospitals in Tigray, 2025.”Prospective follow up study.

PLOS

Point by point response to Reviewers Comments:-

| Sr. No. | Reviewers' Comments | Response to Comments |
| --- | --- | --- |
|  | Academic Editor comments |  |
| 1 | In the abstract should be a short convictive statement showing the identified gap | We accept the comment and we made corrections accordingly. |
| 2 | Please ensure that your manuscript meets PLOS ONE's style requirements, including those for file naming. The PLOS ONE style templates can be found at <https://journals.plos.org/plosone/s/file?id=wjVg/PLOSOne_formatting_sample_main_body.pdf> and <https://journals.plos.org/plosone/s/file?id=ba62/PLOSOne_formatting_sample_title_authors_affiliations.pdf> | We accept the comment and we made corrections according the PLOS ONE guideline. |
| 3 | Your ethical statement should appear at the method section | We accept the and we brings it to the method section |
| 4. | Please include a separate caption for each figure | Thank you for the comment. We have included caption for each separate figures. |
| 5. | We note that you have included a table to which you do not refer in the text of your manuscript | We accept the comment .we have shifted the text toward the table that represented  (Table 4. Results of bivariate and multivariate Cox regression analysis of time to initiate TF and its predictor factor among preterm neonates admitted at public general Hospitals in NICU from December 20,2024 to February 30,2025,Tigray,Ethipia.(n=193) towards the text represents. |
| 6 | Which factors are risk and which are preventive should be concluded. Conclude sound with specific recommendations | Thank you for the comment. Risk factors are : BW < 1500 gm, APGAR scores < 7 (both at 1 and 5 minutes), RDS  Preventable factors are: Key strategies promoting immediate skin-to-skin contact through Kangaroo Mother Care (KMC) and nutritional support protocols for very low birth weight and low APGAR score infants can facilitate a smoother transition to feeding. |
| 7. | Is written informed consent waved or is it signed? It should clearly be stated. If signed who signed the consent? | Written consent was waived from the parent/guardian of the preterm neonate. |
| 8 | Line 62-67-paraphrase the idea | The comment is well taken and we paraphrased the idea. |
| 9. | Please describe, in a succinct way, the presence of feeding protocols at the hospitals? | We accept the comment. Despite the critical need for structured feeding strategies, many hospitals currently lack established protocols for trophic feeding. As a result, the initiation and management of trophic feeding in preterm neonates often vary widely among healthcare providers, leading to inconsistent practices. |
| 10 | Please summarize line 120-123 including reference citations? | We accept the comments and we made summarization on the idea. |
| 11. | The manuscript from line 129 -134 is not convective to conduct this study. Please revise it. What gaps were identified and what was your study’s main aim? | The comment is well taken. The timing of initiating TF in preterm neonates is critical for their health, yet significant gaps exist in understanding this process, especially in general hospitals in Tigray, Ethiopia. Current literature demonstrates limited evidence on the timely initiation of TF among preterm neonates admitted to Neonatal Intensive Care Units (NICUs), and many studies do not utilize a prospective follow-up methodology. This lack of localized data contributes to delays in TF initiation, potentially impacting health outcomes for these vulnerable infants. Identifying specific barriers that affect the timing of TF, such, resource constraints, and the training needs of healthcare providers, is essential for informing improvements in clinical practices. |
| 12. | What other studies found regarding your topic and what is left should be explained? Why providers do not start trophic feeds early despite the evidence available. Also, state how this manuscript will contribute to the evidence available regarding this topic? | Thank you for the nice comment. Despite recognizing the benefits of early TF, healthcare providers may still hesitate to start feeding promptly due to concerns about the stability of the infant, inadequate training in nutritional guidelines, and systemic barriers within the NICU setting. This study aims to investigate the predictors of timely TF initiation among preterm neonates in Tigray and will contribute valuable insights to the existing evidence base. By elucidating the factors that delay TF, the research can provide targeted recommendations to enhance care quality, reduce healthcare costs, and improve long-term health outcomes for families, thereby ensuring that preterm infants receive the critical nutrition they need on time. |
| 13. | Please define pre-diagnosed Stage II or III NEC, Stage III asphyxia and why is this an exclusion criterion? | Pre-diagnosed Stage II or III NEC and Stage III asphyxia are critical exclusion criteria in this study. NEC is a severe gastrointestinal condition predominantly affecting preterm infants, with Stage II indicating moderate disease characterized by abdominal distension and systemic illness, while Stage III represents a more severe form that may involve bowel perforation and increased risk of complications. Stage III asphyxia, or severe hypoxic-ischemic encephalopathy, results from significant oxygen deprivation at birth, leading to severe neurological impairment and clinical instability. Introducing trophic feeding in infants with these conditions could exacerbate their fragile physiological state and increase the risk of serious complications. By excluding these infants, the study can focus on a more homogeneous population of preterm neonates who are better equipped to tolerate early trophic feeding, thus yielding clearer insights into the predictors and benefits of timely TF initiation. |
| 14. | The Methods and materials section does not provide adequate information to replicate the study? | Thank you for the comment. We incorporated the suggestions in the method section. |
| 15 | Simple definitions are not included, like: how are TF provided? | The feeding can be administered via an orogastric or nasogastric tube, depending on the infant's condition and the clinical setting. This method ensures that the feeding is provided directly into the stomach. |
| 16. | Line 227: Survival time is a confusing name for the time to start TF | The comment is well taken and I have replaced with Time to Initiate Trophic Feeding. |
| 17 | Line 234-246 the idea on this text should be clear and summarized: and also please specify/clarify the purpose of the questionnaire; to whom it was administered? This goes accordingly with my comment regarding lack of clarity about the execution of the study? | Primarily used to gather information from respondents and provide standardized method to ask questions, ensuring consistency in response across different participants.  The questions was administered to their parents or guardians. |
| 18. | Please omit line 274-278 | Thank you for the nice comment and we omitted it. |
| 19. | Line 295-310 un-necessary redundant information since the information is provided with Table 1 | Thank you for the nice comment and we omitted it. |
| 20. | Line 350-351 “Kaplan-Meier curves constructed to compare the overall survival patterns over time ‘’for’’ between different groups”. Please omit ‘’for’’ | Thank you for the nice comment and we omitted ‘’for’’. |
| 21 | The discussion is focused on comparing the findings with those noticed in other studies. However, are the findings aligned with international literature? | Thank for the comment.  Yes, we found findings is aligned with international studies. Example, a retrospective follow-up study in Turkey identified KMC as a key factor in enhancing enteral feeding skills among preterm neonates. Additionally, a prospective cohort study conducted in a teaching hospital in India found that early implementation of KMC was safe and correlated with a shorter time to reach full feeds in preterm infants. Furthermore, a meta-analysis of randomized controlled trials indicated that KMC promotes the early initiation of breastfeeding among preterm and low birth weight infants and we included in the manuscript discussion part. |
| 22. | Overall the discussion section needs major revision; does not clearly synthesize your findings and the implication of these findings | We accepted your comments and we made corrections accordingly. |
| 23 | The limitation section should be paraphrased | We accept the comments and we paraphrased to Limitation of this study is the need for continuous monitoring and follow-up. This demand strains resources and necessitates additional time commitments from healthcare providers. |
| 24 | Regarding language: there are English grammatical and syntaxis errors in the whole document. Please ensure that, before resubmission, a person proficient in written English edits the manuscript. | We accepted your comment and we made corrections accordingly across all the manuscript. |

Dear Academic Editor of PLOS ONE,

Thank you for your prompt response and constructive comments
